# The Circulatory and Metabolic Responses to Hypoxia in Humans – With Special Reference to Adipose Tissue Physiology and Obesity

**DOI:** 10.3389/fendo.2016.00116

**Published:** 2016-08-29

**Authors:** Ilkka H. A. Heinonen, Robert Boushel, Kari K. Kalliokoski

**Affiliations:** ^1^Turku PET Centre, University of Turku, Turku, Finland; ^2^Department of Clinical Physiology and Nuclear Medicine, University of Turku, Turku, Finland; ^3^Division of Experimental Cardiology, Thoraxcenter, Erasmus MC, University Medical Center Rotterdam, Rotterdam, Netherlands; ^4^School of Kinesiology, University of British Columbia, Vancouver, BC, Canada

**Keywords:** hypoxia, humans, blood flow, metabolism, adipose tissue

## Abstract

Adipose tissue metabolism and circulation play an important role in human health. It is well-known that adipose tissue mass is increased in response to excess caloric intake leading to obesity and further to local hypoxia and inflammatory signaling. Acute exercise increases blood supply to adipose tissue and mobilization of fat stores for energy. However, acute exercise during systemic hypoxia reduces subcutaneous blood flow in healthy young subjects, but the response in overweight or obese subjects remains to be investigated. Emerging evidence also indicates that exercise training during hypoxic exposure may provide additive benefits with respect to many traditional cardiovascular risk factors as compared to exercise performed in normoxia, but unfavorable effects of hypoxia have also been documented. These topics will be covered in this brief review dealing with hypoxia and adipose tissue physiology.

## General Cardiovascular and Metabolic Responses to Hypoxia

A large body of knowledge on the physiological effects of hypoxia has been obtained over several decades from field experiments in the mountains as well as from studies in environmental chambers, where ambient air is manipulated. Hypoxia, defined as reduced or insufficient oxygen supply caused by reduced oxygen saturation of arterial blood, results in cardiovascular system adjustments to deliver more blood to tissues to compensate for reduced oxygen delivery, which is sensed by oxygen-sensing mechanisms, such as carotid bodies (1). The acute central cardiovascular response to hypoxic stress triggers an increased heart rate at an unchanged stroke volume mediated primarily by increased sympathetic neural discharge as a function of increasing hypoxic severity. At rest, lower levels of hypoxic exposure may result in some degree of systemic vasodilation, while with increasing severity of hypoxia, the peripheral vasculature constricts to redistribute oxygen delivery to the most critically dependent organs, e.g., heart (2–5), brain (6–8) needs to be ensured. This regulation is exacerbated in obstructive sleep apnea, which creates a physiological condition called chronic intermittent hypoxia, which may compromise some functions of the body. Similarly, during exercise in hypoxia, perfusion of skeletal muscle is increased to match oxygen demand, which creates circulatory competition between the locomotor skeletal muscles and other organs, and leads to decreased exercise capacity with severity of hypoxia.

**Figure 1:**
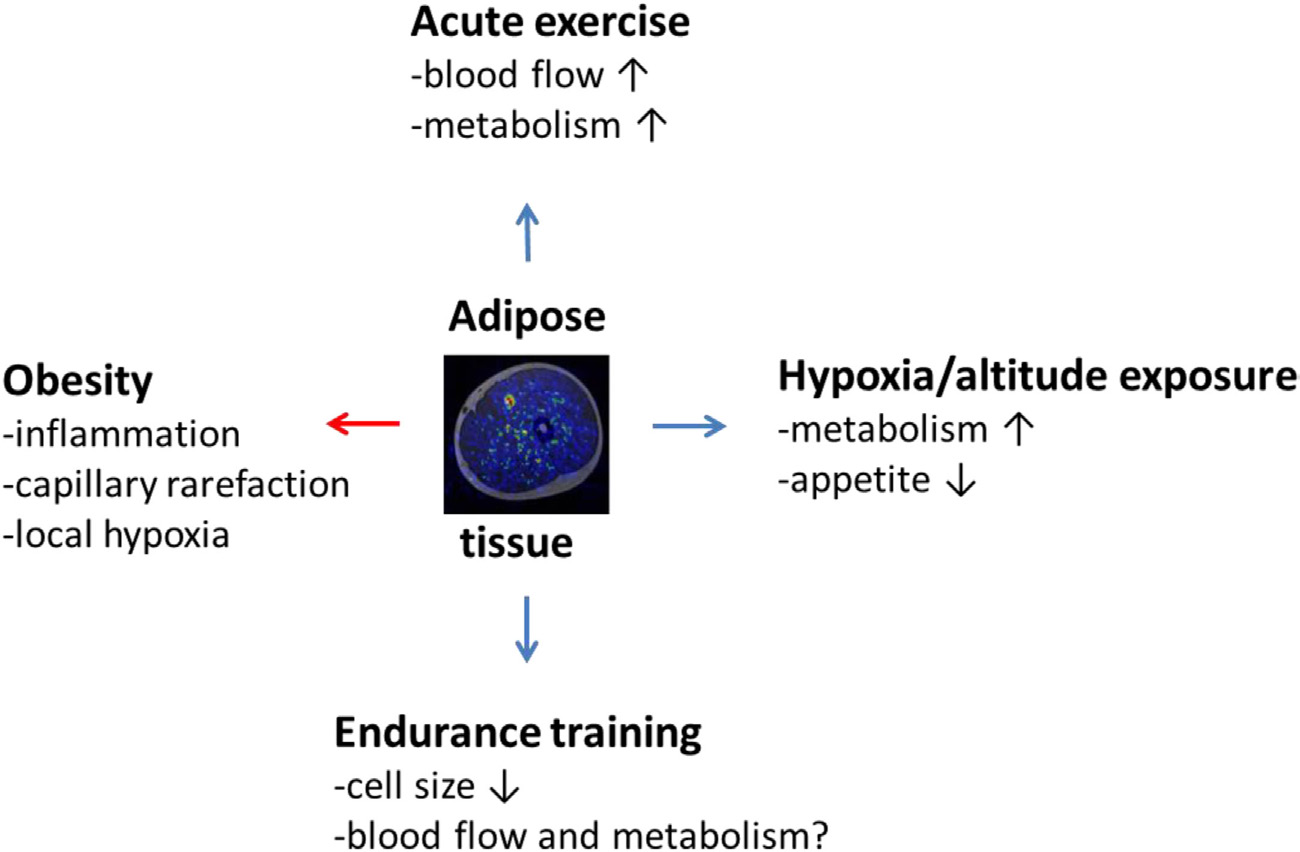
**The effects of obesity, acute exercise, hypoxic or altitude exposure, and endurance training on adipose tissue, such as subcutaneous adipose tissue, surrounding thigh musculature as illustrated by the fusion image in the middle of the figure obtained by combining magnetic resonance and positron emission tomography imaging**. Obesity induces negative (red arrow) inflammatory state in adipose tissue connected with capillary rarefaction and local hypoxia. Acute low or moderate intensity exercise is on the other hand capable of increasing adipose tissue blood flow and metabolism, which, in the long run, reduces adipose tissue cell size and inflammation. This effect may be potentiated by hypoxic exposure or altitude training, but scientific evidence is still in its infancy to prove this hypothesis correct. It also remains to be investigated to what extent classical endurance training can affect adipose tissue blood flow and metabolism in humans.

In addition to cardiovascular stress, hypoxia also alters energy metabolism of the body (Figure 1). Although hypoxia might theoretically even slightly decrease the oxygen requirements at the local tissue level due to reduced oxygen supply, increased sympathetic neural activation and resulting release of various stress hormones often cause whole body metabolism to increase in response to hypoxia (9–14). It has been postulated that, particularly, glucose uptake might be favorably affected by hypoxia (13–17), which has implications for the prevention and treatment of disease states, where metabolism is deranged, such as in diabetes. Not every study, however, supports that view, as decreased skeletal muscle insulin sensitivity (18) and impaired lipid metabolism (19) have also been reported after chronic hypoxic exposures. Hypoxia also alters adipose tissue circulation, which plays an integral role in its metabolism and, therefore, has implications for obesity and diabetes.

## Hypoxia and Adipose Tissue Circulation and Metabolism

Adipose tissue has an important role in regulating metabolism (20–22) – a topic of growing interest as levels of obesity have increased globally over the last several decades. Adipose tissue vasculature and oxygen supply is an important determinant of its metabolism as well as endocrine function (23, 24). Despite the fact that adipose tissue has a capillary surface area less than one-third of that in skeletal muscle (24), it has long been acknowledged that also adipocytes are surrounded by an extensive network of capillaries (23). This vascular feature importantly affects the adaptability of subcutaneous adipose tissue to excess caloric overload, which is known to be associated with a hypoxic state in adipose tissue (25–27). Thus, although opposite views have also been presented (28), it is the common consensus that due to the insufficient blood supply and capillary rarefaction connected with tissue inflammation (20, 25, 29–35), chronic low oxygen levels in expanded adipose tissue is now well appreciated to contribute to metabolic derangements of the whole body.

Although there is also a noticeable extent of variability in physiological responses to hypoxia in humans (36), particularly white adipose tissue is known to respond remarkably to low levels of oxygen. This fact is well illustrated by cell culture studies, where exposure of adipocytes to low oxygen levels alters the gene expression of over 1000 genes (25). However, no change in subcutaneous adipose tissue blood flow is necessarily observed at rest in humans in response to moderate systemic hypoxia (37). Adipose tissue blood flow in humans is under the regulation of the sympathetic nervous system (38), and it is, therefore, reasonable to assume that moderate systemic hypoxia simply does not create a high enough stimulus for sympathetic neural vasoconstrictor activation to reduce blood flow in healthy human adipose tissue. On the other hand, it is also plausible that the activation of vasoconstriction by arterial chemoreceptors predominates over a local hypoxic vasodilation in adipose tissue in humans. In this regard, adipose tissue appears to be similar to bone (39). However, increased blood flow in response to systemic hypoxia has been documented in human skin (40). More studies are clearly warranted to explore whether unchanged hypoxic blood flow is also of importance to explain pathophysiological characteristics of adipose tissue under chronically low oxygen levels that is not compensated by increased blood flow (20, 25).

It is known that subcutaneous adipose tissue blood flow increases in response to low intensity exercise, but levels off when exercise intensity is further increased (41). Furthermore, at rest, but not during exercise, subcutaneous adipose blood flow is under the control of nitric oxide (42). In contrast to resting conditions, subcutaneous adipose blood flow is reduced during exercise, when subjects breathe hypoxic air (37). This novel finding is likely based on the constriction of adipose tissue vasculature by hypoxia-triggered enhanced sympathetic nervous system activity, which redistributes limb blood flow to exercising muscles, which depend more critically on adequate oxygen supply in response to exercise. In this regard, we have previously reported that blood flow in subcutaneous adipose tissue is significantly lowered by local infusion of norepinephrine, which is the principal neurotransmitter released from the sympathetic nerve endings, and that the inhibition of α-adrenergic receptors by phentolamine tends to enhance adipose tissue blood flow, both at rest and during exercise (38). It has also been previously suggested by Romijn et al. (43) that the reduction of adipose tissue blood flow is likely to be one important mechanism to explain decreased free-fatty acid release in response to high intensity exercise, which then leads to preferential utilization of glucose instead of fatty acids and contributes to the increased efficiency of ATP generation for a limited O_2_ availability. Additionally, it has been recently documented that the inability to increase vascular resistance in adipose tissue during exercise or to maintain mean arterial pressure during orthostatic stress in aging is largely a result of reduced α-adrenergic responsiveness of adipose tissue arterioles (44, 45). Therefore, it is concluded that reduced blood flow in adipose tissue is an acute physiological response to diminished oxygen availability during exercise, while higher blood flow in adipose tissue is needed in response to prolonged exercise that also likely associates with higher lipolysis to supply more free-fatty acids into circulation to sustain muscular work for prolonged periods (46).

In addition to the general hypoxic responses, the capacity of blood flow in human subcutaneous adipose tissue has remained largely unexplored, until recently. In this regard, a novel finding is that the vasodilatory capacity of human subcutaneous adipose tissue determined by infusion of exogenous dilator compounds approaches the physiological level reached during moderate intensity exercise (37). Furthermore, during this maximal vasodilation, vascular conductance can reach a level even higher than that induced by exercise. In terms of absolute values, the comparison of adipose tissue blood flow capacity to skeletal muscle is also of interest. In this regard, we have previously reported that blood flow in human skeletal muscle during a similar pharmacological vasodilation protocol increases to a level of 40 ml/min/100 g (47). As the absolute average value of pharmacologically induced adipose tissue blood flow was 10.5 ml/min/100 g, it only reaches ~26% of blood flow level in the muscle. Accordingly, the functional vascular capacity appears to be very closely followed by that of structural anatomy, as adipose tissue is known to have a capillary surface area that is slightly less than one-third than that in skeletal muscle (24). In relative terms, blood flow in adipose tissue increased 8-fold and blood flow in muscle 14-fold in response to pharmacological (adenosine) infusion, and, as such, the increase in adipose tissue flow is 57% of that of muscle. In contrast to human skeletal muscle (47), pharmacologically induced blood flow is not, however, positively and significantly related to subjects’ whole body maximal oxygen consumption determined in a separate fitness test, indicating that blood flow in adipose tissue and muscle do not simply parallel each other. Nevertheless, it can be concluded, based on these studies, that the functional blood flow capacity of adipose tissue is fairly large in healthy human subjects. It remains, however, to be measured if this capacity is lost in pathological states. Furthermore, it also remains to be determined if a loss of functional vascular capacity is linked to impaired fat storage in white adipose tissue which is known to contribute to metabolic and cardiovascular derangements in a human body (20).

## Hypoxia as a Treatment of Obesity and Impaired Adipose Tissue Physiology?

As summarized in the beginning of the previous section, it is evident that there is a hypoxic state in adipose tissue of obese subjects, which may be caused by insufficient circulatory responses/adaptations in response to lowered oxygen supply. Despite this, chronic and/or intermittent hypoxia has also been suggested as treatment option for overweight and obesity (9, 10). This is based on findings that hypoxia alters the function of the nervous system and hormonal levels such as leptin, which lead to changes in glucose metabolism and control of appetite (9–14). These physiological responses are enhanced with increasing severity of hypoxia, such as altitude exposure. There is evidence that people living at high altitude are less likely to be overweight and/or obese, the findings which hold after adjustment for many plausible confounding factors that might also affect the association (48, 49). Protective effects of hypoxia/altitude have also been reported in regards to development of diabetes (50) and coronary heart disease, as well as stroke (51–53), meaning that hypoxia reduced the incidence of these diseases (Table 1). Furthermore, interventional trials have been conducted to test the effects of hypoxia as a treatment for weight loss and improvement of metabolic functions (Table 1). These studies demonstrated that 7 h of moderate hypoxia under resting conditions did not change postprandial glucose responses or substrate oxidation in young healthy men (54). However, when hypoxic exposure was combined with low intensity physical activity, Netzer and colleagues reported greater weight loss in obese subjects when compared to combined exercise and sham hypoxia intervention (55), although this finding could not be reproduced in their recent study (56). Beneficial effects of hypoxia regarding body weight control have also been reported in obese young adults (57). Furthermore, Haufe et al. comprehensively investigated numerous cardiovascular risk factors in response to hypoxic training and showed favorable influences on body fat content, triglycerides, fasting insulin, and insulin sensitivity, as compared to exercise training only intervention (58). These findings were confirmed in their later study in overweight and obese men with lower exercise workload, which reduces exercise burden for overweight subjects and is, thus, beneficial in terms of exercise compliance (59). Appetite regulation is not necessarily always affected, although lipid profile is improved (60). Altogether, it is concluded that training in hypoxia appears to have numerous additive and favorable effects on traditional cardiovascular risk factors, which may have important clinical implications (61, 62).

**Table 1:** **Summary of studies investigating the effects of hypoxia on cardiovascular and metabolic health in humans**.

Reference	Type of the study	Outcome(s)
Voss et al. (48)	Epidemiological	Lower rates of new obesity diagnoses among overweight persons at high altitude
Voss et al. (49)	Epidemiological	Obesity prevalence inversely associated with elevation and urbanization
Woolcott et al. (50)	Epidemiological	Inverse association between diabetes and altitude
Ezzati et al. (51)	Epidemiological	Living at higher altitude had a protective effect on ischemic heart disease and a harmful effect on chronic obstructive pulmonary disease. No net effect on life expectancy or associations with stroke and cancer after adjustments for confounders
Faeh et al. (52)	Epidemiological	Linearly decreased ischemic heart disease mortality with increasing altitude
Faeh et al. (53)	Epidemiological	Lower mortality from coronary heart disease and stroke at higher altitudes
Morishima and Goto (54)	Acute 7 h experimental trial at rest	No effect of hypoxia on postprandial glucose responses or substrate oxidation in young healthy men
Netzer et al. (55)	Exercise training in normobaric hypoxia	Significantly greater weight loss in obese persons in real hypoxia than in sham hypoxia
Gatterer et al. (56)	A randomized, single blind, placebo-controlled study	No larger reductions in body weight due to moderate intensity exercise and rest in hypoxia compared to normoxia alone in obese subjects
Kong et al. (57)	Experimental trial	Normobaric hypoxia training caused more weight loss than normoxia training in obese young adults
Haufe et al. (58)	Single blind exercise training under hypoxia or normoxia	Endurance training in hypoxia resulted in a similar or even better response in terms of cardiovascular and metabolic risk factors than endurance exercise in normoxia
Wiesner et al. (59)	Single blind exercise training under hypoxia or normoxia	Training in hypoxia elicited a similar or even better response in terms of physical fitness, metabolic risk markers, and body composition at a lower workload in obese subjects
Debevec et al. (60)	Hypoxic confinement at simulated altitude with and without daily moderate intensity exercise	Body mass decreased in both groups, but whole body fat mass was only reduced in the exercise group. No change in hormonal appetite regulation, but improved lipid profile due to combined training and hypoxia exposure
Bailey et al. (61)	4-day experimental trial	An additive cardioprotective effect of normobaric hypoxia training over training in normoxia
Wee and Climstein (62)	A review of 25 hypoxic training trials	Hypoxic training may be beneficial as an adjunct treatment to modify some cardiometabolic risk factors

Despite plausible physiological mechanisms and some encouraging results that hypoxia might indeed work as a potential therapeutic tool to tackle obesity, it may also have detrimental influences that need some consideration. First, hypoxia might not be well-tolerated by all subjects, as high-altitude illness is experienced in approximately 10 to 25% of unacclimatized persons above 2500 m, and the prevalence and severity of symptoms increases with increments in altitude (63). Second, hypoxia is associated with impaired cognitive performance, which may persist even after the cessation of hypoxic exposure (64). Third, hypoxic exposure is known to impair human immune system function (65), which may be detrimental in fighting against pathogens and other triggers of communicable diseases. Fourth, hypoxia is capable of inducing fibrosis in cardiac muscle (66), which increases the stiffness of the heart. Many obese individuals already have cardiac stiffness (67, 68), which may be exaggerated by hypoxic exposure. Finally, as mentioned in the previous section, hypoxia triggers an inflammatory response in adipose tissue of obese subjects, which may be further exacerbated by hypoxia creating a vicious-cycle. Thus, hypoxia not only alters human energy metabolism, which may lead to weight loss if not compensated for by increased energy intake, but is also capable of inducing several physiologically detrimental effects on bodily functions. It is likely that the balance of all these determine the overall outcome and health effects of hypoxia in humans.

Finally, as hypoxia is indeed a common feature of adipose tissue in particular, and potentially other tissues in obese subjects, it has been suggested that hyperoxia might be an option to overcome the hypoxic state. However, as oxygen is known to be toxic in high concentrations, this treatment may not be healthy in terms of circulatory and metabolic function. Hyperoxia is known to decrease adipose cell viability, increase both intra- and extracellular oxidative stress, provoke inflammation, and decrease glucose uptake of adipocytes (69). Hence, based on this information on hyperoxia and reviewed knowledge regarding hypoxia, it is concluded that there is a delicate balance of healthy oxygen supply and demand in adipose tissue that determines its overall function. While mild hypoxia over a sufficient duration of exposure may provide some additional benefits, the most feasible approach to address obesity and individual weight loss appears to reside in more traditional methods proven to be efficient in reducing adipose tissue size: physical activity and diet rich in fruits and vegetables, but low in caloric energy (Figure 1).

## Author Contributions

IH drafted the manuscript and all authors contributed to its revision and intellectual content.

## Conflict of Interest Statement

The authors declare that the research was conducted in the absence of any commercial or financial relationships that could be construed as a potential conflict of interest. The reviewer MH and handling Editor declared their shared affiliation, and the handling Editor states that the process nevertheless met the standards of a fair and objective review.
